# Meeting August 2

**Published:** 1886-09

**Authors:** 


### CHICAGO MEDICAL SOCIETY.

*Regular Meeting, August 2, 1886.*—THE PRESIDENT, E. J. DOERING, M. D., in the chair.

DR. L. L. MCARTHUR, read a paper on

DIAGNOSIS AND TREATMENT OF HEPATIC ABSCESS, which appears in another part of the *Journal and Examiner*.

DR. FRANK BILLINGS upon invitation said he thought that probably he had had less experience with living patients with abscess of the liver than any one present, and consequently had less that is practical to say about it. While in Vienna he saw a great number of autopsies of abscesses of the liver. Several cases occurred in the wards, but no diagnosis of abscess was made, the patients suffering from what appeared to be intermittent fever, and usually a diagnosis of that kind was made. A curious thing to him was the fact that a great number of these abscesses were found in new-born children. In vienna, one often sees in the dead-house abscesses which were the result of badly attended cord. Pus had formed in the vein and the inflammation spread upward, finally producing abscess in the liver.

PROFESSOR H. A. JOHNSON said, that while listening to this paper, a somewhat unusual case was recalled to his mind which occurred at the county hospital when it was located on eighteenth street. It was a case of abscess in the left lobe of the liver which had communicated, evidently some time before death, with the left lung, and was discharged through the bronchus; when the case came into the hospital it presented all the signs of empyema and the complication of the abscess with the liver was only discovered in the dead-house. Another case occurred to him in which there was abscess in the right lobe of the liver discharging through the right lung. The patient recovered, but five or six years afterwards a recurrence of the abscess took place, and it discharged into the vena cava, producing instant death. Post-mortem revealed the cavity of the abscess communicating with the vena cava. Both of these cases seemed to him to be somewhat out of the ordinary history of abscesses of the liver.

DR. J. J. M. ANGEAR said, that in all cases that he had seen the discharge had a fearful odor, and he began to think that if the pus did not have that odor, he should have doubts of its being an abscess of the liver.

DR. J. A. ROBISON said that he had just had in the county hospital a case of abscess of the liver in which the diagnosis was made only after death. The remark of Dr. Billings, that abscess of the liver is often diagnosticated as malaria, led him to speak of it. The case was that of a woman who had been ill for several months. When he first saw her in the hospital about a month ago, she was greatly emaciated, jaundiced, and complaining a great deal of pain in the hypogastric region. The liver was not enlarged, her temperature ranged from 100° F. to 102° F., she had chills every few days, and it was supposed they were due to malaria. She finally died of exhaustion.

There was no diarrhœa, but there was great tenderness over the hypogastric region, together with general jaundice. There was also a cachectic appearance which led him to believe there might be catarrhal inflammation of the duodenum, which would account for the jaundice. But upon post-mortem examination it was found to be a case of multiple abscesses of the liver, with purulent inflammation of the gall ducts. He had in the same hospital another case in which nearly all the signs, of which Dr. McArthur has spoken here, occurred. The patient was a woman about 40 years of age, in whom the area of liver-dulness was greatly increased. Her temperature was 100° F., she was extremely jaundiced, and when she first came into the hospital he heard the friction sound as mentioned in the paper, but that has again disappeared. He believed the case would prove to be one of large abscess of the liver.

DR. JOHN BARTLETT said that at the time of the Mexican War, many of the soldiers returned affected with chronic diarrhœa. In seven of these cases which came under his observation, enormous abscesses of the liver followed. They were not opened, but were particularly examined in the dead-room, some of them containing at least a gallon of pus. As a general rule the patients lived for months. About a year ago he was called to see a case in consultation. The man had been trodden upon in a crowd and received a slight injury about the epigastrium. Inflammation followed and abscess of the liver was diagnosticated, but owing to a difference of opinion among the consultants no effort was made even to aspirate. After death, which followed in due course, multiple abscesses were found, one as large as a small cocoanut, another as large as an orange, and fifty other smaller ones. He mentioned this to prove how hopeless would have been an attempt to improve the condition of this man by aspiration. He saw another case of some interest: The man was shot and some weeks afterward died. The body was opened and a piece of wadding and the bullet were found in the liver. The abscess was about as large as two fists and opened into the vena porta. In another case he was called in consultation to see a man who appeared to have an affection of the liver. Both of the attending physicians thought it was a case of abscess. Presently he had symptoms indicating that there was some difficulty in the respiratory organs, and later, but in time to act if they had been more prompt, they discovered that there was a retropharyngeal abscess. He immediately expressed the opinion that the abscess in the liver had opened into the anterior mediastinum. He deemed it inexpedient to open this abscess without having other instruments than they had with them. They went to get others and when they returned they were advised at the gate that man had been taken with a difficulty of breathing which simulated croup and had immediately expired. Upon examination they found that he had been drowned, as it were, by the pus from the bursting of this large abscess.

DR. H. N. MOYER asked Dr. MacArthur if in the literature on the subject he had seen anything in reference to the presence of peptone in the urine in these cases? Jaffey has laid down indications of diagnostic value when this substance is formed He thought the symptoms might lead to the development of this substance in the urine, and that it might be of some diagnostic value, in suspected cases of abscess of the liver.

DR. MACARTHUR said, in closing the discussion, he was interested to learn of another source of abscess of the liver to be by infection from the umbilical cord as related by Dr. Billings. Dr. Johnson's case of abscess of the liver breaking into the left pleura and left lung is the only one of which he had known. Rupture into the vena cava is also rare. To Dr. Angear he would say that in the cases he saw the discharge was odorless, and he had emphasized that fact in the paper because the books all state that as a rule it possesses a very offensive odor, and is usually of a purulent character. In two of the cases which he saw it was of a chocolate color being mixed with blood and liver-tissue. Dr. Robison had spoken of a case as markedly jaundiced. This is set down as an exceedingly rare complication in abscess of the liver. He said that in that case the liver was found, post-mortem, to contain multiple abscesses. He desired to ask if the cause was found? Dr. Robison replied that none was found. He also wished to ask Dr. Bartlett if his case proved to be a case of abscess of liver with a mediastinal opening? Dr. Bartlett said that they presumed it to be hepatic, but no post-mortem was allowed. As to peptone, he had seen no literature on the subject in connection with the liver. In those cases in which he used the hypodermic needle to make a diagnosis, three in number, two were operated on and recovered, the third was treated by the expectant treatment and died. No careful search was made for the puncture of the needle in the abscess of the liver. After the first two cases he became bold in the use of the hypodermic needle and punctured for the fourth time in one case before he obtained purulent matter.

DR. SCOTT HELM read a paper entitled

A SUBCUTANEOUS METHOD OF THE TREATMENT OF BUBOES, WITH EXHIBITION OF THE INJECTING INSTRUMENT.

This paper appears in full in another part of the *Journal and Examiner*.

His method consists in injecting the suppurating gland, after the pus has been withdrawn, a solution of carbolic acid to wash out the cavity, and then injecting and allowing to remain an emulsion of iodol in pure oleic acid. The injecting instrument consists of a barrel holding two drams, which is mounted on either side by two rings for the fore and middle fingers, and a ring in the end of the piston for the thumb. Three needles, two different sizes of aspirator needles, and one a canula with trocar. To these is attached the center joint, in which is a stôpcock, the opposite extremity of which is attached by a smooth joint to the barrel. In twenty-three cases the treatment was successful in all but the nineteenth, this patient having gone on a protracted spree following the operation. The advantages of the operation on that when there are two or more suppurating buboes in the same chain of lymphatics, the second or third appear further away from the initial one; by placing the first glandular abscess in a perfectly aseptic condition you prevent the inflammations of neighboring glands; secondly, there is no cicatrix remaining.

DR. J. ZEISLER said that if conservative surgery has any place he thinks it is in the treatment of buboes, and he thinks the final bad result of treating buboes by section might be avoided by carrying out the idea of Dr. Helm. It is a good idea to inject iodol, as it may be regarded as a specific against the venereal poisons, and there can be hardly a doubt that the suppuration is due generally to the poisons. He would like to know if Dr. MacArthur had found it sufficient to introduce this iodol emulsion more than once?

PROFESSOR G. C. PAOLI said that in the treatment of buboes of course the sooner we get out the pus the better, but those who have had experience with the treatment of buboes know that there are cases in which, in spite of aspirations and subcutaneous injections, there is still a morbid process going on which produces mischief and ulceration of the tissues. He never uses carbolic acid, but he has used a watery solution of permanganate of potash one-half grain to six ounces, injecting it into the cavity. But we all know that we have cases of buboes which, in spite of all the skill of the physician, produce great suffering. He had seen a case where the femoral artery had to be ligated. In temperate persons we have very tedious buboes. Again, where mercury has been used too freely there are often mischievous buboes. Very difficult cases are those in business men who are busy, active, and produce more congestion of the abscesses by their activity. But if we can get the patient to go to bed and rest we succeed better in our treatment. However he thinks favorably of the subcutaneous method.

DR. FRANK asked Dr. Helm how many of these buboes were due to gonorrhœa and how many to chancres or chancroids, if any?

DR. H. N. PIERCE said he had had an opportunity of seeing Dr. Agnew, Jr., four years ago, experiment with the subcutaneous treatment of buboes. He first evacuated the pus by use of the canula, then injected an antiseptic solution, washing it out and afterward applying a compress bandage. The buboes generally went on from bad to worse, and he tried the same method over again but with no success, and finally he had to cut down upon them and treat them by the old method.

DR. HELM, in closing the discussion, said that he had made, in any case, only one injection, and the time that elapsed before the patient was discharged had been from eight to fourteen days. In reply to Dr. Feder he would say that these buboes had been aspirated as early as possible, as soon as there was fluctuation. All the pus was removed and the cavity injected with a like quantity of the oleic acid emulsion of iodoform. He had no case in which there had been a return of the disease in the same chain of glands. He had one case in which there was a bubo appeared some two weeks afterward in the opposite groin, evidently due to gonorrhœa. In the second case, and the last one, which had only occurred a few days ago and was not reported, the buboes were caused by gonorrhœa; all the others were due to chancroids.

IN MEMORIAM.

Since our last meeting it had pleased Divine Providence to call from our midst Dr. Robert C. Hamill, and the Committee on Necrology submitted the following resolutions:

*Resolved*, that this Society has learned with profound sorrow of the death of Dr. Hamill, one of the earlier as well as one of the most earnest and efficient members of this Society.

*Resolved*, that in the death of Dr. Hamill this Society has lost a member typical of the true gentleman in the kindness of his manner, in the great force and energy of his character; typical of the good citizen in his ceaseless efforts to advance the interests of institutions tending to ameliorate the condition of the sick and unfortunate; typical of the patriot in his unceasing efforts to aid and cherish the disabled soldiery of his country; typical of the wise counsellor in the soundness of his judgments; typical of the true physician in his earnest cultivation of medical knowledge and in his philanthropic practice ol the healing art; typical of the faithful Christian in his never flagging zeal in all good works.

*Resolved*, that the Society extend to the bereaved widow of the deceased, in her great loss, sympathy and condolence.

JOHN BARTLETT,
CHAS. GILMAN SMITH,
H. A. JOHNSON,
*Committee on Necrology.*

